# Electrical Bioimpedance Analysis: A New Method in Cervical Cancer Screening

**DOI:** 10.1155/2015/636075

**Published:** 2015-02-02

**Authors:** Lopamudra Das, Soumen Das, Jyotirmoy Chatterjee

**Affiliations:** ^1^School of Medical Science & Technology, Indian Institute of Technology, Kharagpur 721302, India; ^2^Department of Material Engineering, Indian Institute of Science, Bangalore 560012, India

## Abstract

Cervical cancer is the second most common female cancer worldwide and a disease of concern due to its high rate of incidence of about 500,000 women annually and is responsible for about 280,000 deaths in a year. The mortality and morbidity of cervical cancer are reduced through mass screening via Pap smear, but this technique suffers from very high false negativity of around 30% to 40% and hence the sensitivity of this technique is not more than 60%. Electrical bioimpedance study employing cytosensors over a frequency range offers instantaneous and quantitative means to monitor cellular events and is an upcoming technique in real time to classify cells as normal and abnormal ones. This technology is exploited for label-free detection of diseases by identifying and measuring nonbiological parameters of the cell which may carry the disease signature.

## 1. Introduction

Cancer of the cervix uteri is one of the most common cancer in women with an estimate of 527,624 new cases and 265,653 deaths in 2012 [1]. The mortality rates of cervical cancer worldwide are significantly lower than its rate of incidence. The mortality to incidence ratio of the disease is reported to be 50.3% [1]. The squamous cell carcinomas of the uterine cervix are more prevalent compared to the adenocarcinomas [2]. Conventional Pap smear is the most common and widely used technique for screening cervical cancer. But this technique suffers from a very high false negativity of about 40%. The liquid based cytology though improved the sensitivity of Pap screening is too costly to be employed in the developing countries [3, 4]. So effort has been made in the present study to understand the biophysical signatures during health and disease which may be helpful to detect the disease at a much earlier stage before any alteration of the cytomorphology can be noted [5, 6]. Electrical characterization of cervical tissue has been proposed as a tool to improve the sensibility and specificity of cervical cancer screening [7–10]. A fascinating and an extremely interesting area of research is the study of bioelectrical properties of cells, the biological entity. This method of cell study has proved its potential in extracting data about the morphology and physiology of the cells [11]. This technology identifies and measures the nonbiological parameters of the cells which may bear the disease signature and can be used for label-free disease detection. A cell when subjected to an electric field offers resistance to the current flow and shows its bioimpedance characteristics. The insulating properties of the living cells are different under different applied frequency [12]. In order to sustain the required potential difference the cells provides varying resistance and capacitance [7]. The cellular impedance varies for different cellular activities in static and dynamic conditions. Thus the frequency response of the electrical bioimpedance of the biological cells and/or tissues is greatly influenced by their physiological and physiochemical status and is different from subject to subject. Even the complex bioelectrical impedance varies within tissues in a particular subject and also differs with the change in its health status [13, 14] depending on the physiological and physiochemical changes of the tissues health. This biological phenomenon provides a variable, real time, probe-free, highly sensitive, cost effective, spatiotemporal monitoring option for automated analysis of cellular behaviour in vitro [15].

In studying of cellular electrical property, cytosensors are used [16]. Impedance cytosensor provides real time and quantitative means to study cellular events, such as changes of ionic channels as well as membrane integrity, cell spreading, motility, and growth [17, 18] and to detect analytes by converting cellular responses into a measurable electrical signal [19, 20]. Impedance cytosensors have also been employed to detect and monitor apoptosis induced changes in cells [21–23]. A preliminary study has already been reported of being conducted on Columbian setting for the detection of cervical cancer [10]. This upcoming technique of bioelectrical property study in real time may be valuable in classifying cells as normal and abnormal ones in cancer screening.

Hence, the present study aims at electrical characterization of cervical exfoliative cytology in suspension for classifying them as normal and abnormal ones in the screening process via a fast and real time bioimpedance analysis technique.

## 2. Materials and Methods

### 2.1. Study Sample

A total of 150 samples were collected under the informed consent of patients and ethical clearance from 150 women for the electrical bioimpedance study of the cervical smear in suspension between the periods of April 2011 and August 2013. The samples were collected from women with mean age of the volunteers being 54.6 years. The study was conducted in full accordance with the ethical principles and guidelines of Medical Council of India including the World Medical Association Declaration of Helsinki. Out of 150 cases, the number of abnormal cases was 23 of which 8 were atypical squamous cells of undetermined significance (ASCUS), 9 low-grade squamous intraepithelial lesions (LSIL), and 6 high-grade squamous intraepithelial lesions (HSIL).

### 2.2. Sample Collection

The samples were collected from patients by means of Ayer's spatula and/or endocervical brush. The sampling devices collect the samples covering different zones of the cervix and the cells were transferred into the vials labelled with patients' identification number and containing an isotonic salt solution to retain the intact cellular status for bioimpedance analysis.

### 2.3. Bioimpedance Measurement

**Figure 1:**
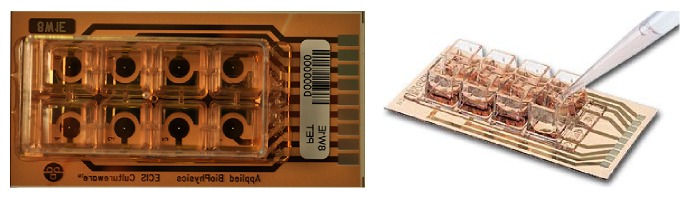
Specialized 8-well ECIS device.

The cervical cells in the polysol solution were subsequently vortexed for 15 seconds and the cell suspension was centrifuged at 1700 RPM for 3 min. The supernatant was discarded and the cell pellet was resuspended in 500 *µ*L of fresh polysol solution. The bioimpedance of the cell suspension was measured using commercially available miniature planar electrode (model number 8W1E), impedance based device (Applied Biophysics, USA) as shown in Figure 1, and LCR meter (HOIKI, Japan). 500 *µ*L volume of the cell suspension containing 10^4^ cells/mL was poured in the device for bioimpedance measurement.

The samples were subjected to the frequency sweep of 100 Hz to 1 MHz using AC voltage signal of 10 mV peak to peak and LCR meter was employed to measure the corresponding impedance. There were 26 measured data points over the whole range of frequency sweep. The bioimpedance of the cells was measured for 127 normal and 23 abnormal cases. The frequency response characteristic of bioimpedance was plotted using Origin 8 software by considering the average impedance values against each of the 26 measured frequencies for all the normal and abnormal cases separately along with their standard deviation.

## 3. Result

### 3.1. Bioimpedance Analysis of Cervical Smear

**Figure 2:**
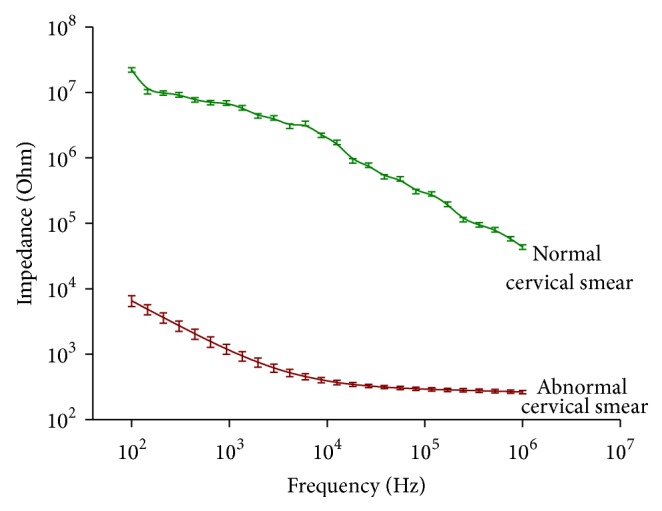
Variation of bioimpedance along with the standard deviation of normal and abnormal cervical cells over the electrical frequency range of 100 Hz to 1 MHz.

Bioimpedance characteristic was plotted against the frequency range for both the normal and abnormal cervical smear and is produced as Figure 2. The bioimpedance of the normal samples was found to be three orders higher in contrast to that of the abnormal ones over the whole frequency range of 100 Hz to 1 MHz. Moreover, a steady decrease in bioimpedance characteristic was noted in the graph with increasing frequencies for the normal samples (Figure 2) whereas, in case of the abnormal ones, a fall in the impedance value was observed till the frequency of 10^4^ Hz and reached saturation thereafter for the higher frequency.

The measured results of the electrical properties of the cervical cells in suspension clearly depicted that the electrical signatures of the normal cells were distinctly different from that of the abnormal samples. From the graph (Figure 2) it was also evident that the bioimpedance of the normal cells was much higher compared to the abnormal one.

## 4. Discussion

**Figure 3:**
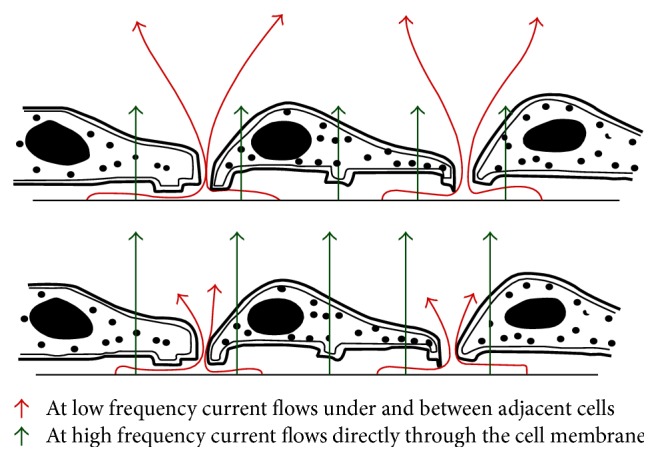
Schematic representation of the current flow path at low and high frequencies.

Detecting the disease at its early stage is the key to reduce the mortality rates in malignancies [24, 25] and cytopathological screening has a leading role in it [26, 27]. In case of malignancy of the uterine cervix, routinely used conventional Pap smear (CPS) suffers from about 30 to 40% of false negative rates [27] mainly due to cell crowding and superimposition of inflammatory cells, presence of RBC, and other allied artefacts. So for minimising the high rates of false negativity of cervical screening, an effort has been made in the present study to explore the possibility of bioelectrical property study of cervical cells in suspension. The novelty of this work lies in the combinatorial approach of biophysical attribute such as bioimpedance study along with the existing cytological examination which may yield a meaningful and precise data towards dropping down the false negative rates. Moreover such attempt has been made for the first time to evaluate the bioelectrical property of the cells along with its morphological features to increase the sensitivity of the available screening technique and also for detecting the disease at an early stage. Alteration in biophysical attribute such as bioimpedance may be noted much prior to any observable morphological change [20]. The presence of different ion channels with specific selectivity and permeability accounts for the voltage gradient across the plasma membrane [28] and this potential difference across the membrane plays a crucial role in cellular proliferation and differentiation [29]. The bioelectrical properties in case of cancer cells are different due to loss of membrane heterogeneity and thus the depolarised voltage across the membrane favours cell proliferation and migration [28]. The alteration in the ion channels in course of disease progression may contribute to the change of biophysical signatures [28]. Figure 2 represents the bioimpedance attribute pattern of normal and abnormal cervical smear over the frequency range of 100 Hz to 1 MHz. From the preliminary study, it may be clearly noted that the impedance characteristic of the normal cervical cells is about three orders higher compared to the abnormal ones over the entire range of frequencies. It may be noted that N/C ratio is low for normal compared to abnormal cells implicating larger nucleus size in abnormal cells [30]. The literature survey indicated [7, 12, 15–17] that, with increasing frequency, the impedance magnitude drops and it holds well in case of our data also. Moreover the trends of this obtained data also match the study conducted on the Colombian setting for the detection of the cervical cancer [10]. The cell membranes exhibit dielectric property at lower frequencies resisting the current flow through it. This may be due to the fact that, according to Figure 3, at low frequencies (<2,000 Hz) the impedance values are higher because most of the current flows in the solution channels under and between adjacent cells (red arrows) (Figure 3) which may be due to the dielectric property of the cell membrane to resists the flow of electrical current through the membrane at lower frequencies. But at higher frequency the current passes through the cells due to lower reactance of the plasma membrane. But at higher frequency (>40,000 HZ) more current capacitively couples directly through the insulating cell membranes (green arrows) (Figure 3) due to lower reactance of the plasma membrane resulting in lower impedance values.

So, accounting for the above-mentioned factors, it is usually expected that the flow of current will mostly occur through the nucleus in case of abnormal samples illustrating much lower impedance of the cells, attributed to noncompact DNA organisation within the nucleus. With the onset and progression of cancer, alteration in the ion channels occurred leading to the variation of membrane potential [31]. But the ion channels present at cell membrane in normal samples are generally dedicated for a specific process of ion transportation and thus render higher cytoplasmic resistivity. The electrical conduction path is greater in cytoplasm compared to its nucleus and thus the resistance offered by cytoplasm will also be much higher as represented in Figure 2. The experimental findings principally indicate the alteration of cellular bioelectrical properties due to change in its physiological attributes during disease progression. However detailed and quantitative analysis is required to implement this approach towards clinical screening process.

## 5. Conclusion

For minimisation of false negativity in the cervical cancer screening with Papanicolaou test (Pap smear), there is a need to explore novel cytological technique as well as identification of unique and important cellular features from the perspectives of morphological, biomolecular, or biophysical properties. In the present study biophysical parameters such as electrical bioimpedance analysis were performed and the electrical bioimpedance analysis of cervical cells evidenced the difference between the abnormal and normal conditions. In future, combining of bioimpedance data along with the existing cytopathological examination may increase the efficiency of the cervical screening to a greater extent thereby reducing the rates of faulty diagnosis.
